# Metagenomic and ribosomal transcript profiles of diabetic foot osteomyelitis in Hispanic patients: underestimated bacteria in biofilm persistence

**DOI:** 10.3389/fcimb.2025.1729196

**Published:** 2026-01-28

**Authors:** Leonor Díaz-Velis, Francisco Salvador-Sagüez, Freddy Roach, Edgardo Mancilla, Marco A. Campos, Tay Ruiz-Gil, Mateo López-Moral, Gabino Garrido, José Luis Lázaro-Martínez

**Affiliations:** 1 Laboratory of Applied Microbiology Research (LIMA), Faculty of Health Sciences, Universidad Católica de Temuco, Temuco, Chile; 2 Molecular Ecology and Applied Microbiology Laboratory, Department of Pharmaceutical Sciences, Universidad Catolica del Norte, Antofagasta, Chile; 3 Infectious Diseases Unit, Dr. Leonardo Guzmán Regional Hospital, Antofagasta, Chile; 4 Department of Medical Sciences, Faculty of Medicine and Dentistry, Universidad de Antofagasta, Antofagasta, Chile; 5 Microbiology Area, Clinical Laboratory, Dr. Leonardo Guzmán Regional Hospital, Antofagasta, Chile; 6 Pathology Unit, Dr. Leonardo Guzmán Regional Hospital, Antofagasta, Chile; 7 Centro de Investigación, Innovación y Creación, Vicerrectoría de Investigación y Posgrado, Universidad Católica de Temuco, Temuco, Chile; 8 Diabetic Foot Unit, Complutense University of Madrid, Madrid, Spain; 9 Independent Researcher, Antofagasta, Chile

**Keywords:** 16S rRNA gene sequencing, bone microbiome, diabetic foot osteomyelitis, metagenomics, polymicrobial infections, ribosomal transcript analysis, biofilm

## Abstract

**Background:**

Diabetic foot osteomyelitis (DFO) is a serious complication of diabetes and a leading cause of lower-limb amputations. Conventional culture-based diagnostics often underestimate the microbial diversity of infected bone tissue. This study represents the first characterization of both total and ribosomally active bone microbiota in Hispanic patients with DFO using high-throughput *16S* rRNA gene sequencing. The work aims to contribute to the inclusion of underrepresented populations in microbiome research and informing molecular-based antimicrobial strategies.

**Methods:**

Bone specimens (n = 13) were collected from seven Chilean patients with histologically confirmed DFO. Samples were analyzed using conventional aerobic culture and *16S* rRNA gene sequencing from both genomic DNA (gDNA) and complementary DNA (cDNA) to characterize the total bacterial community and the ribosomally active fraction. In three patients, samples were stratified by bone depth (superficial/top, middle and bottom). Microbial diversity and relative abundance were assessed across patients and bone layers.

**Results:**

Acute osteomyelitis was the predominant histopathological pattern. Culture yielded 19 bacterial isolates, 95% of which were Gram-negative bacilli. Sequencing identified 3,412 operational taxonomic units (OTUs), with Proteobacteria, Bacteroidetes, Firmicutes, and Actinobacteria as dominant phyla. Enterobacteriaceae and Enterococcaceae were the most ribosomally active families. Microbial community composition varied substantially among patients and across bone depths. *Staphylococcus aureus* was infrequent (5% of culture isolates; ~1% of sequence reads), whereas low-abundance but ribosomally active taxa, such as Corynebacteriaceae, were consistently detected across all layers.

**Discussion:**

This combined metagenomic and ribosomal transcript analysis reveals a polymicrobial, patient-specific bone microbiota in Chilean patients with DFO, highlighting potentially active bacteria frequently overlooked by standard diagnostic methods. These findings underscore the value of integrating molecular approaches into clinical workflows to improve pathogen detection and support more personalized antimicrobial strategies, while also helping to address gaps in microbiome research among underrepresented populations.

## 1 Introduction

Diabetes is a multifactorial metabolic disease affecting over 537 million adults worldwide and costing approximately $966 billion in health expenditure. Its prevalence is projected to reach 643 million people by 2030 (Tönnies et al., 2021). In Latin America, Chile reports the highest diabetes rates, with 12.3% of individuals over 15 and 30.6% over 65 years old affected (Margozzini and Passi, 2018). Among diabetic patients, the risk of developing a lower-extremity ulcers ranges from 19% to 34%, and non-healing ulcers precede ∼85% of non-traumatic amputations (Yazdanpanah et al., 2015).

Diabetic foot osteomyelitis (DFO), a bone infection in diabetic patients, accounts for about 15% of moderate and up to 50% of severe infections (as defined by the International Working Group on the Diabetic Foot/Infectious Diseases Society of America, IWGDF/IDSA), often leading to minor and/or major amputations (Giurato et al., 2017). Five-year survival after such amputations ranges from 41% to 48% (Lin et al., 2020). Determining the risk factors for amputation in diabetes with an early and accurate diagnosis is key to preventing recurrence and reducing the rate of amputation.

Microbiological culture is the standard method used to guide antibiotic treatment; as it identifies cultured microbes and determines antibiotic sensitivity. However, its sensitivity is limited, particularly in bone infections. Studies report low concordance between surface wound cultures and bone isolates, although *Staphylococcus aureus* remains the most common pathogen (Díaz-Velis et al., 2023). DFO is a polymicrobial infection, dominated by *S. aureus* (>50% of cases), with frequent presence of *Staphylococcus epidermidis*, Streptococcus, Enterobacteriaceae and less abundant Gram-negative and anaerobes (Charles et al., 2015; Shettigar et al., 2018; Choi et al., 2019; Macdonald et al., 2021; Villa et al., 2024). However, more than 99% of microbial species remain uncultured, the uncultured microorganisms named the “dark matter”, could potentially explain therapeutic failure in DFO (Bernard et al., 2018).

Chronic osteomyelitis has a high recurrence rate despite prolonged antibiotic therapy (Acharya et al., 2013; Game, 2013). Incomplete surgical debridement may lead to biofilm formation (Ruppé et al., 2017). Culture-independent techniques like high-throughput sequencing (HTS) offer deeper insights, identifying unculturable and transcriptionally active microbes (Lam et al., 2016; van Asten et al., 2016; Ruppé et al., 2017; Jneid et al., 2018; Kalan et al., 2019; MacDonald et al., 2019; Schmidt et al., 2021; Schmidt, 2022). Metagenomic analysis is carried out on genomic DNA isolated from the environment under study, but it does not distinguish whether this genomic DNA comes from cells that are viable or not or whether the predicted genes are actually expressed and under what conditions (Gosalbes et al., 2011). In this regard, transcriptomics has been gaining popularity, specially metatranscriptomics, for functional insights. Characterizing the phylogenetic structure of the ribosomally active bacterial fraction through the analysis of 16S ribosomal RNA transcripts provides an approach to identify the bacteria potentially participating in the community at the time of sampling, offering a more dynamic view of the microbial community (Bashiardes et al., 2016). Unfortunately, all omics-based studies in DFO have focused on populations of European or North American ancestry. This lack of representation hinders the generalizability of microbiome-based findings to the global population and risks perpetuating health disparities, especially in regions such as Latin America, where the prevalence of diabetes continues to rise.

In this context, our study aimed to characterize both culturable and non-culturable microbiota in DFO bone tissue using conventional culture and Illumina-based *16S* rRNA sequencing of genomic DNA and cDNA. We assessed total and ribosomally active bacterial communities and, for the first time, analyzed the microbiota stratified by bone depth in a Hispanic population. Our findings offer novel insights into the ribosomal active bacteria potentially driving infection persistence and treatment failure.

## 2 Materials and methods

### 2.1 Patient recruitment and sample collection

This observational study included seven Chilean patients (six men and one woman; P1-P7) with a mean age of 61 years, all diagnosed with DFO (IDSA 3, SEWSS, moderate PEDIS). None had received antibiotics in the two weeks prior to sampling. After providing informed consent approved by the Scientific Ethics Committee of Universidad Católica del Norte and Hospital Regional de Antofagasta (HRA), patients underwent bone biopsy in a secondary-level procedure room or private clinic’s surgical pavilion, following the protocol by Féron et al. (2021) (Féron et al., 2021).

Under sterile conditions, bone samples were taken from the exposed lesion site (**Table 1**). In patients P3, P4 and P7 -who had more severe lesions- samples were collected in three layers (top, middle, bottom; approximately 3 x 4 mm in size). A single sample was taken from patients P1, P2, P5 and P6 due to less extensive involvement.

**Table 1:** Clinical and histopathological characteristics by patient and sample.

Sample ID		P1	P2	P3			P4			P5	P6	P7			Media	SD
				Top	Middle	Bottom	Top	Middle	Bottom			Top	Middle	Bottom		
Clinical parameters	Age (Years)	58	67	69			59			69	55	65			63,1	5,7
	Sex	M	M	M			F			M	M	M			-	-
	Diabetes mellitus (Years)	15	>15	>15			>15			12	10	>15			12,3	2,5
	Glycated hemoglobin (%)	9,4	8,3	8,3			7,3			7,3	10,4	8,6			8,5	1,1
	White blood cell count (10e9/L)	7200	9800	6900			8600			7400	5700	12400			8285,7	2231,9
	ESR (mL/h)	53	70	10			81			13	72	45			49,1	28,4
	GFR (mL/min)	72	33	81			99			73	103	124			83,6	29,0
Histopatology	Ulcer location	Left calcaneus	Residual phalanx of the 3rd toe	5th metatarsal of the left foot			Lateral border of the 3rd toe			Head of the 5th metatarsal, plantar side	Fibular malleolus	Right foot, diaphysis of the first metatarsal			-	-
	Osteomyelitis type	CO	AO/ACO/CO	ACO	ACO	ACO	AO	AO	AO	CO	AO/AO/AO	AO	AO	AO	-	-
	Matrix	Fibrosis	Osteopenia/Remodeling/Remodeling	Fibrosis	Fibrosis	Fragmentation	Osteopenia	Osteopenia	Osteopenia	Fibrosis	Fragmentation/Fragmentation/Fragmentation	Osteopenia	Fragmentation	Fragmentation	-	-
	Tissue vitality	Vital	Osteonecrosis/Osteonecrosis/Vital	Osteonecrosis	Osteonecrosis	Osteonecrosis	Osteonecrosis	Osteonecrosis	Osteonecrosis	Vital	Osteonecrosis/Osteonecrosis/Osteonecrosis	Osteonecrosis	Osteonecrosis	Osteonecrosis	-	-
	Cells	Plasmocytes	Neutrophils/Neutrophils/Plasmocytes	Neutrophils	Neutrophils	Neutrophils	Neutrophils	Neutrophils	Neutrophils	Plasnocytes	Neutrophils/Neutrophils/Neutrophils	Neutrophils	Neutrophils	Neutrophils	-	-
	Others	-	Bacteria/-/-	Bacteria	Bacteria	Bacteria	-	-	-	-	Bacteria/Bacteria/Bacteria	Bacteria	Bacteria	Bacteria	-	-
Microbiology	Clinical isolates	*P. stuarti + S. aureus*	*P. mirabilis*	*P. aeruginosa*	*P. aeruginosa*	*P. aeruginosa*	*P. mirabilis + E. cloacae*	*P. mirabilis + E. cloacae*	*P. mirabilis + E. cloacae*	*M. morganii*	*P. mirabilis + E. coli*	Negativo a las 48 h de incubación	*P. aeruginosa + E. cloacae*	*P. putida + E. cloacae*	-	-
	Treatment time (Months)	11	36	7			15			4	6	6			12,1	11,2
	Heealing time (Months)	16	N/A	9			11			4	12	N/A			-	-
	Oclussive artherial disease	negative	negative	positive			Positive			positive	negative	positive			-	-
	Neuropathy	positive	positive	negative			Positive			positive	positive	positive			-	-
	Post-treatment amputation	negative	positive (major amputation)	negative			Negative			negative	negative	positive (minor amputation)			-	-
	Surviving time (Years)	4	3	>5			5			4	4	>5			-	-
	Cause of death	Aspiration Pneumonia By Stroke	Sepsis by DFO	Alive			Cardiopulmonary Arrest			COVID 19	COVID 19	Alive			-	-

Summary of clinical parameters including time of living with diabetes, admission laboratory values, glycosylated hemoglobin (HbA1c), erythrocyte sedimentation rate (ESR), and glomerular filtration rate (GFR, renal function). Histopathological features include findings from anatomical pathology and conventional microbiology, as well as duration of infection, vascular status, and presence of neuropathy.

*DFO, Diabetic Foot Osteomyelitis.

†N/A, Not applicable.

‡CO, Chronic osteomyelitis.

§ACO, Active chronic osteomyelitis.

||AO, Acute osteomyelitis.

Each sample was divided into three subsamples: one for bacterial culture (in a sterile 0.9% saline solution and sent to the HRA microbiology lab), one for anatomical and pathological analysis (fixed in 10% formalin and processed by the pathology lab) and one for bacterial community analysis (preserved in RNALater™ stabilization solution, ThermoFisher, USA and stored at -80°C for DNA/RNA extraction).

### 2.2 Histopathological analysis

Subsamples (single or triplicate per patient) were assessed according to each center´s protocols. Fixation occurred within 24–48 hours, followed by 72 hours EDTA-decalcification. Standard processing produced paraffin-embedded blocks. Serial sectioning (3-4 μm thick) was stained with hematoxylin and eosin, and examined under a light microscope by a single pathologist.

Histological diagnosis was categorized as:

- Acute osteomyelitis: predominance of polymorphonuclear neutrophils in bone marrow.
- Chronic osteomyelitis: lymphocytic infiltrate with plasma cells.
- Chronic active osteomyelitis: equal mixture of both components.

### 2.3 Analysis of cultured bacteria

Upon arrival at the HRA microbiology lab, subsamples were processed immediately. After confirming they met acceptance criteria, each was numbered consecutively. In a biosafety cabinet under sterile conditions, subsamples were homogenized in brain-heart infusion (BHI) broth using one of two methods: (a) large fragments were cut with bone pliers and immersed in BHI; (b) smaller samples were directly immersed in BHI within their transport containers. All samples were vortexed for 10 seconds and incubated (37°C, 12–18 hours) (Burillo et al., 2007).

Post-incubation, the homogenates were plated on blood, chocolate, and MacConkey agar using sterile swabs and loops. Blood and chocolate agar were incubated (37 ± 2°C, 5% CO_2_ for 18–24 hours); MacConkey agar was incubated aerobically for the same duration. Plates without growth were incubated up to 48 hours.

All colonies were considered significant and underwent Gram staining. Identification and susceptibility testing were performed with the Vitek^®^2 system, following CLSI 2016 guidelines. In rare or ambiguous phenotypes, the Kirby-Bauer method and/or Epsilon test were used (Bauer et al., 1959; Parada et al., 2016).

Anaerobic bacteria were not assessed, as this was not part of the standard procedures at HRA.

### 2.4 Nucleic acids extraction and sequencing of the *16S* rRNA gene V4–V5 region

A total of 13 subsamples were processed in 2 analytical groups: One single subsample from each P1, P2, P5 and P6 was used for both gDNA and RNA extraction, while single subsamples from the top, middle and bottom layers from P3, P4 and P7 were used only for gDNA extraction. The gDNA and RNA extractions were performed using PowerSoil DNA and RNA isolation kits (MO BIO Laboratories Inc., CA, USA) following the manufacturer’s instructions. RNA was reversed-transcribed into cDNA using the High-Capacity cDNA Reverse Transcription Kit (Applied Biosystems) from 1 µg/µL of total RNA per subsample, to profile the ribosomally active bacterial community.

The qualities of gDNA and cDNA were assessed using the Qubit dsDNA HS assay (Invitrogen, CA, USA). PCR amplification of the *16S* rRNA gene using universal primers 27F (forward; 5′-AGAGTTTGATCATGGCTCAG-3′) and 1492R (reverse; 5′-GGTTACCTTGTTACGACTT-3′) was performed to verify template integrity. Products visualized on 2% agarose gels stained with ethidium bromide under UV light, with *Escherichia coli 16S* DNA as a control.

For profiling the total bacterial communities and ribosomally active fraction, the V4–V5 regions of the *16S* rRNA gene were amplified from gDNA and cDNA, respectively, using primers 515F-Y (5′-GTGYCAGCMGCCGCGGTAA-3′) and 926R (5′-CCGYCAATTYMTTTRAGTTT-3′) (Parada et al., 2016). Amplicons were sequenced (2×301 bp, paired-end) on a MiSeq platform at Macrogen Inc. (Seoul, Republic of Korea), following the manufacturer’s protocols.

### 2.5 Sequence analysis

Raw data results were processed using Mothur pipeline. Rare sequences differing by 1–2 base pairs were preclusted and chimeras were removed using VSEARCH (RRID: SCR_016964) (Schloss et al., 2009). Taxonomic classification was performed, non-bacterial sequences were removed, and OTUs were calculated at a distance of 0.03 using cluster.split command. For family-level taxonomic distribution (P1–P7), OTUs with identical taxonomic classification were retained as distinct units.

Sequences (from Macrogen) were analyzed with Mothur 1.39.5 and the SILVA NGS reference database (v132: 152308 Bacteria, 3901 Archaea and 16209 Eukarya sequences; RRID: SCR_006423). Data were processed according to the MiSeq SOP protocol. Richness and diversity metrics-including Observed OTUs (Subject observation index, S_obs_), Chao1, Shannon and Simpson indices-were calculated and singletons and chimeras were excluded. Relative bacterial abundances at the phylum, family and genus levels were calculated for both total (gDNA) and ribosomal active (cDNA) bacterial communities.

To generate microbial interaction networks,.shared and.taxonomy files from Mothur (RRID: SCR_011947) were converted into Cytoscape-compatible format using a modified RStudio script (RRID: SCR_000432) (Neave et al., 2014). Nodes represented samples and total OTUs per sample; edges were assigned as distances between the OTU node and sample. The network was visualized in Cytoscape v.3.5.0 (RRID: SCR_003032) (Shannon et al., 2003) using the Force Atlas Algorithm.

For final microbial network, Gephi software (RRID: SCR_017284) was used with the following Force Atlas parameters: inertia, 0.1; repulsion force, 100.0; attraction force, 100.0; autostabilization (force, 100.0; sensitivity, 0.2); gravity, 55.0; attraction distribution, 1.0; speed, 1.0. Node sizes were adjusted to highlight sample sources.

## 3 Results

### 3.1 Clinical data and histopathological analysis of patient samples

The patients included in this study were aged between 55 and 69 years, comprising six men and one woman. All patients had a history of diabetes mellitus lasting more than 10 years and, at the time of bone sampling, presented with an average glycated hemoglobin (HbA1c) level of 8.5 ± 1.1%. Renal function was impaired in all cases, with one patient diagnosed with renal failure. The average duration of OPD treatment was 12 months, except for patient P2, who received intermittent antibiotic treatment for 36 months until his death following a major amputation. This patient was the only one to die from complications related to diabetic foot over the eight-year follow-up period. All remaining patients had a survival period of more than four years. Five of them died during the COVID-19 pandemic: two due to virus-associated pneumonia and two from cardiovascular causes (stroke and cardiopulmonary arrest). One patient died after three years as a result of sepsis secondary to a diabetic foot infection, also associated with a major amputation (**Table 1**). While some of patients were within the high-risk age groups for COVID-19 and had significant cardiovascular risk factors, the deaths observed in the study were unrelated to the underlying DFO or the successful outpatient treatment received. These deaths did not affect the study outcomes because the metagenomic and ribosomal activity analyses were conducted on samples obtained prior to these clinical events.

In five out of the seven patients, three histological samples were obtained from different bone depth layers (P2–P4, P6, and P7). Acute osteomyelitis was diagnosed in four of these patients, based on suppuration due to neutrophilic infiltration, the presence of pyocytes or necrotic debris, osteonecrosis, and visible bacteria. These patients also exhibited osteopenia, fibrosis, or bone fragmentation. Two patients presented with chronic osteomyelitis, characterized by bone marrow fibrosis and plasma cell infiltration, without visible bacterial presence (**Table 1**).

### 3.2 Bacterial isolation in diabetic foot osteomyelitis

A total of 19 bacterial strains were isolated from 13 bone samples collected from seven patients with histologically confirmed DFO. These strains corresponded to eight different bacterial species. Of the isolates, 95% were Gram-negative bacilli, with the most prevalent being Proteus mirabilis (26.3%; 5 isolates), Enterobacter cloacae (26.3%; 5 isolates), and *Pseudomonas aeruginosa* (21.1%; 4 isolates). Less frequent species included *Providencia stuartii*, *Escherichia coli*, *Morganella morganii* and *Pseudomonas putida* (each 5.3%; 1 isolate). The remaining 5% corresponded to a single Gram-positive coccus, *Staphylococcus aureus* (**Table 2**). In 38% (5/13) of the samples, only one bacterial species was identified. In 54% (7/13), two species were isolated. One sample showed no bacterial growth after 48 hours of incubation.

**Table 2:** Bacterial isolates cultured from bone samples of patients with diabetic foot osteomyelitis (DFO).

Bacteria	Species	Number of isolates (N = 12*)	RA% (N = 19)
Gram-negative bacilli	*Proteus mirabilis*	5	26,3%
	*Enterobacter cloacae*	5	26,3%
	*Pseudomonas aeruginosa*	4	21,1%
	*Providencia stuartii*	1	5,3%
	*Escherichia coli*	1	5,3%
	*Morganella morgani*	1	5,3%
	*Pseudomonas putida*	1	5,3%
Gram-positive cocci	*Staphylococcus aureus*	1	5,3%
Total		19	100%

*One of the thirteen samples showed not bacterial growth after 48 hours of incubation.

†RA, relative abundance, expressed as a percentage of the total number isolates.

### 3.3 Richness and diversity of total bacterial community and ribosomally active bacteria

From *16S* rRNA gene sequencing of both genomic DNA (gDNA) and complementary DNA (cDNA) derived from patient bone samples, a total of 2,073,017 reads and 3,412 operational taxonomic units (OTUs) were identified. Sample coverage ranged from 0.98 to 0.99, ensuring reliable detection of taxa and accurate estimates of richness and diversity (**Supplementary Table S1**).

**Figure 1:**
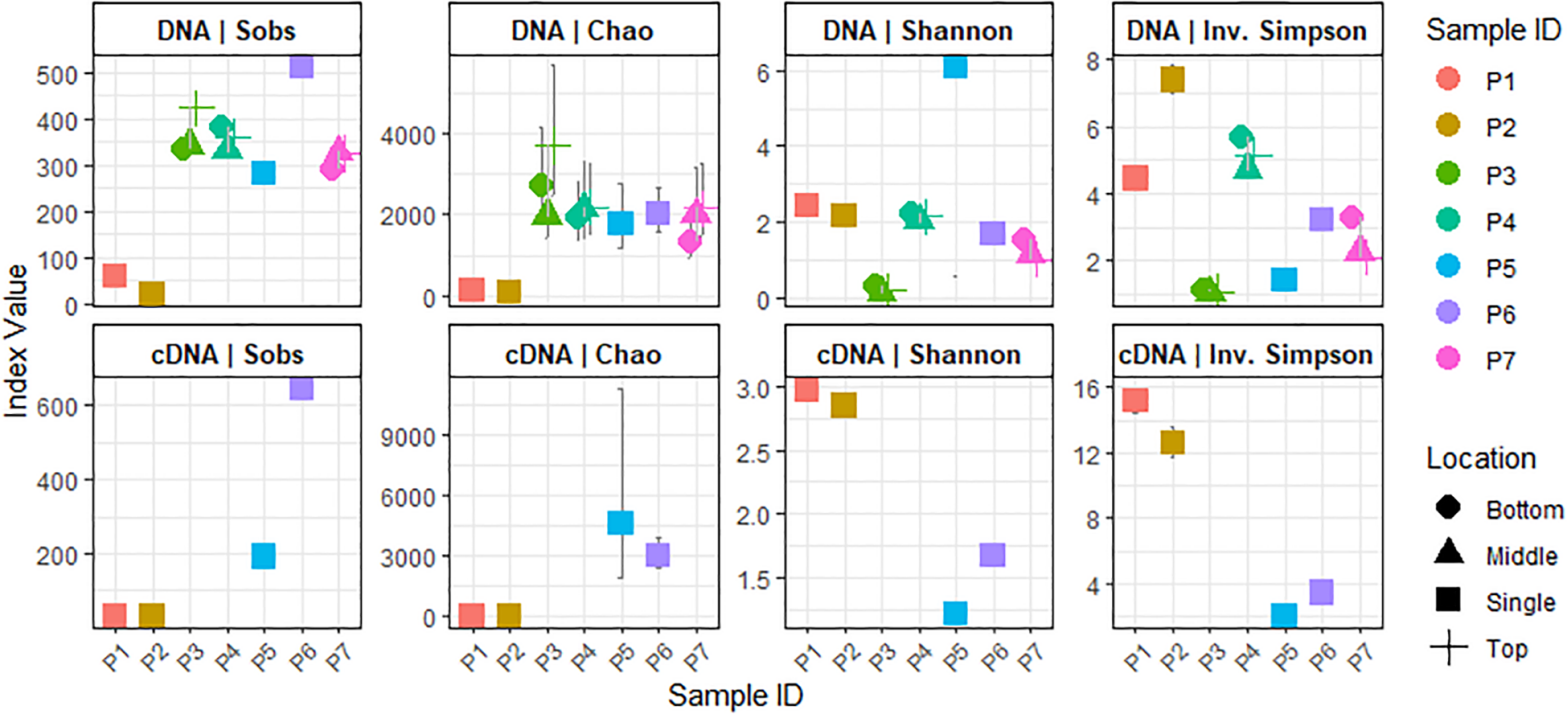
Richness and diversity indices of total and ribosomally active bacterial community in DFO. Samples P1, P2, P5, and P6 are single samples, one per patient. Samples P3, P4, and P7: three layers of bone tissue (Superficial or Top, Middle and Bottom).

Richness scores varied significantly among samples and sequencing type. For the total bacterial community (gDNA), the observed OTUs (S_obs_ index) ranged from 62 to 515. Higher richness was observed in samples P3, P4, P5, P6, and P7 (282–515 OTUs), while P1 and P2 had lower values (22–62 OTUs). The Chao1 estimator showed similar trends, with values ranging from 1351.67 to 30,709.15 for P3–P7 and from 77 to 130 for P1 and P2. Ribosomal transcript–based bacterial communities (cDNA) followed the same pattern (**Figure 1**).

Shannon and Simpson diversity indices revealed that the highest bacterial diversity was observed in all P4 samples (Shannon: 2.03–5.67), followed by samples P1, P2, P5, P6, and all P7 samples (1.01–6.12). P3 samples exhibited the lowest diversity (Shannon index from 1.25 × 10^-7^ to 1.13). Interestingly, P1 and P2 showed higher ribosomal activity (Simpson index: 2.97–15.59) compared to P5 and P6 (1.22–3.4) (**Figure 1**).

### 3.4 Total bacterial community and ribosomally active bacterial composition per patient with DFO

**Figure 2:**
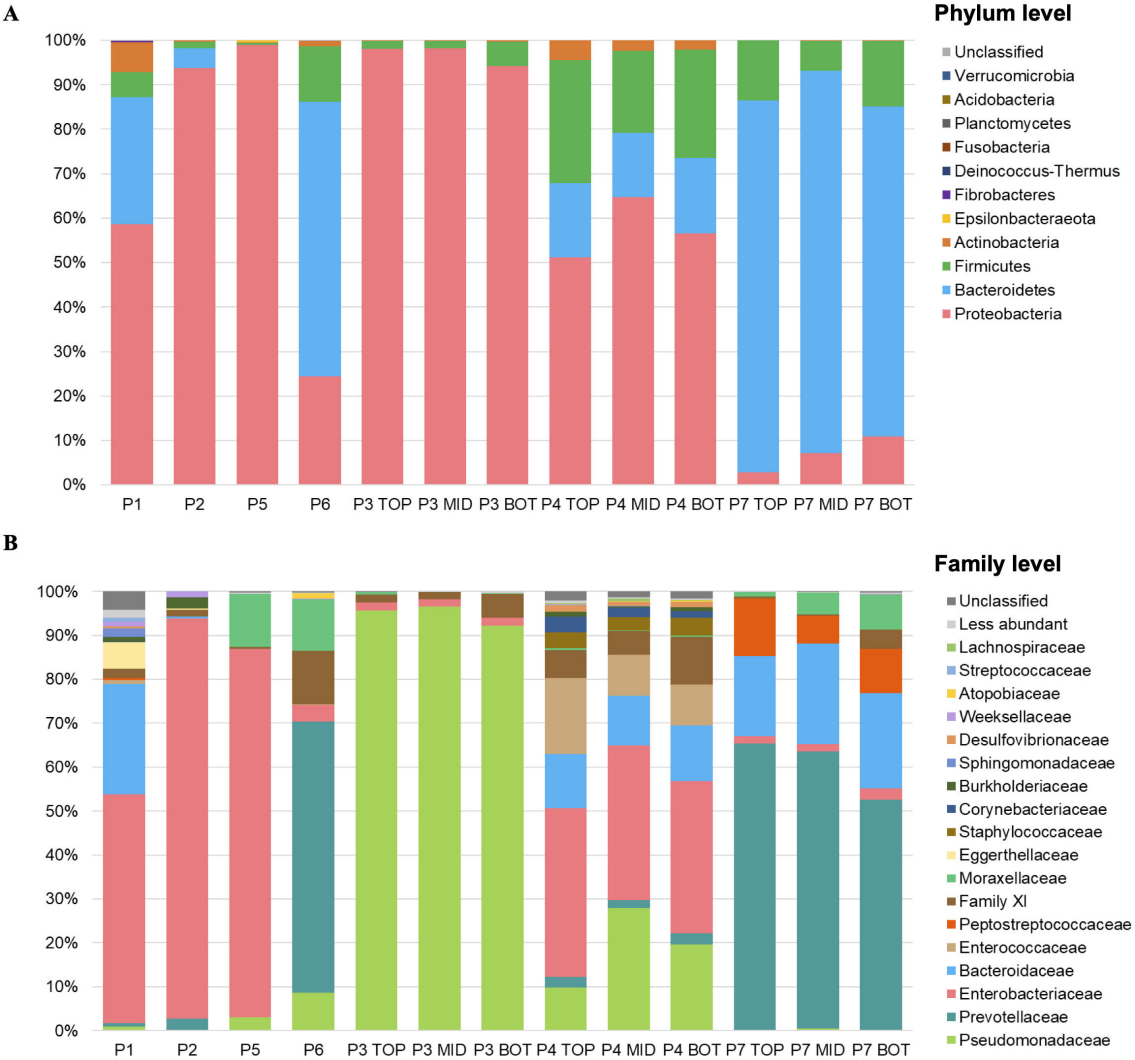
Relative abundance at **(a)** phylum level and **(b)** family level for total bacterial community of the seven patients with DFO. Samples P1, P2, P5, and P6 are single samples, one per patient. Samples P3, P4, and P7: three layers of bone tissue: Superficial or Top, Middle and Bottom.

The identified OTUs were classified into 12 phyla, 11 classes, 91 families, and 118 bacterial genera. At the phylum level, the most abundant taxa across all samples were Proteobacteria (58.4%), Bacteroidetes (29.8%), Firmicutes (10.3%), and Actinobacteria (≥0.1%) (**Figure 2A**). At the family level (**Figure 2B**), the top ten most abundant families were Pseudomonadaceae (27.3%), Enterobacteriaceae (26.9%), Prevotellaceae (19.5%), Bacteroidaceae (9.6%), Family_XI (3.0%), Moraxellaceae (4.5%), Enterococcaceae (2.8%), Peptostreptococcaceae (2.3%), Staphylococcaceae (0.8%) and Corynebacteriaceae (0.6%).

**Figure 3:**
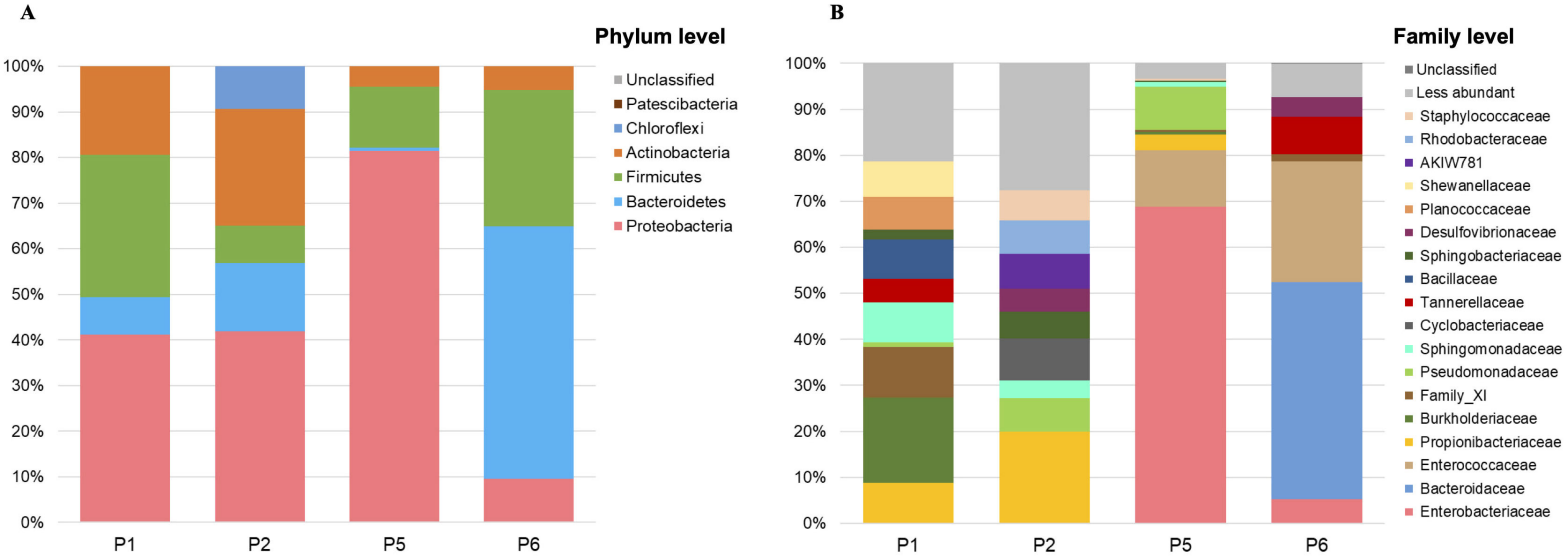
Relative abundance at **(a)** Phylum level and **(b)** Family level for ribosomally bacterial community of the four patients with DFO. Samples P1, P2, P5, and P6 are single samples, one per patient.

For the total ribosomally active bacterial community, the dominant phyla were similar: Proteobacteria (43.5%), Firmicutes (20.7%), Bacteroidetes (19.8%), Actinobacteria (13.6%), and Chloroflexi (9.4%) (**Figure 3A**). The most abundant families included Enterobacteriaceae (18.5%), Bacteroidaceae (11.8%), Enterococcaceae (9.6%), Propionibacteriaceae (8.1%), Burkholderiaceae (4.6%), and others (**Figure 3B**).

**Figure 4:**
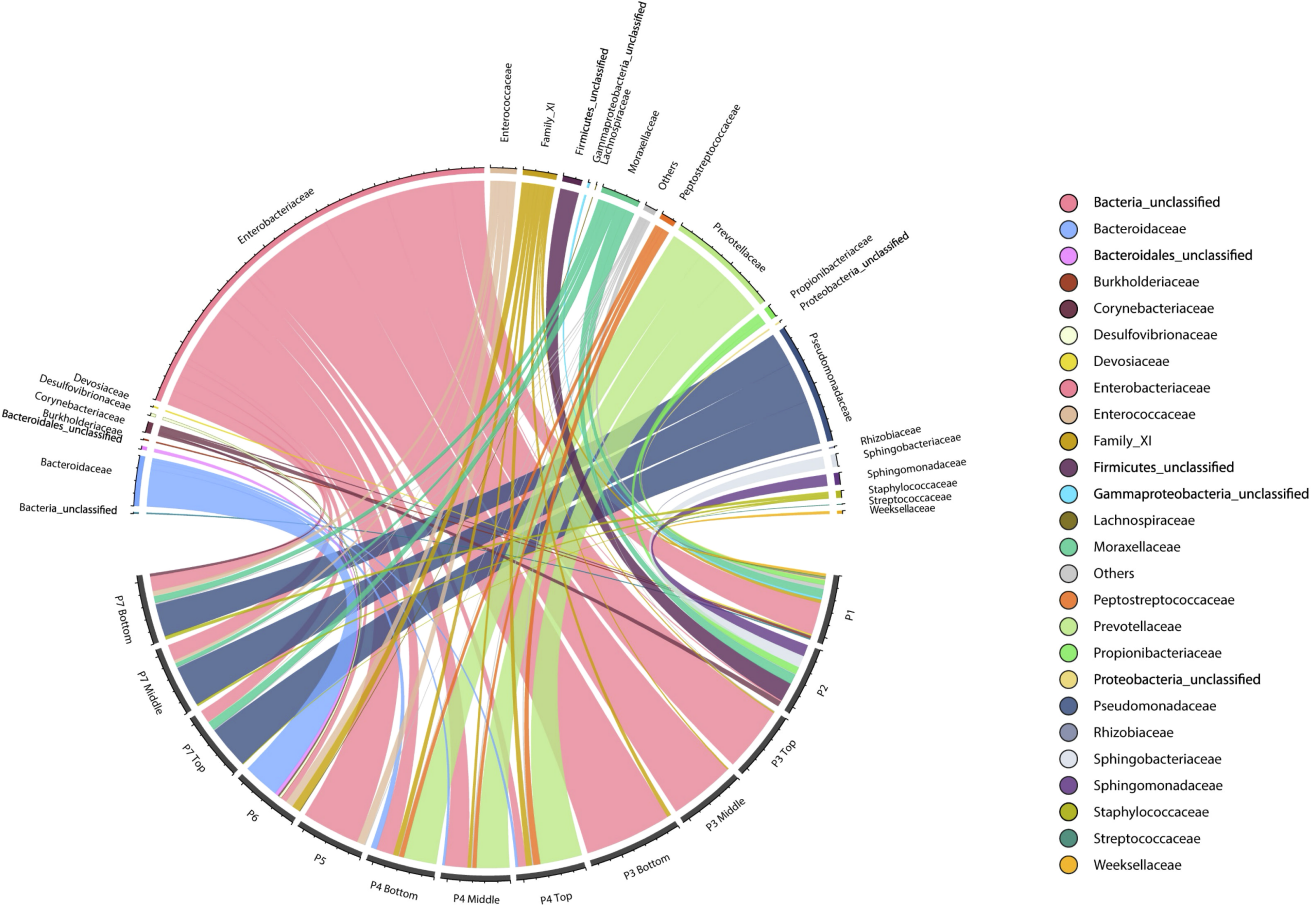
Taxonomic distribution of OTUs at the family level in diabetic foot osteomyelitis. For each patient (P1–P7), different OTUs assigned to the same taxonomic classification were maintained as independent OTUs.

The total bacterial composition for each patient was determined at the taxonomic family level, 16S rDNA-based analysis of DFO samples revealed a diverse bacterial community representing the total microbiota, including viable, nonviable and dormant bacteria. Enterobacteriaceae was the only family detected in all samples, with highly variable relative abundances (1.6%–91.1%). The dominant family varied by patient, for example: Prevotellaceae dominated P6 (61.8%) and all P7 depths (60.3%), Enterobacteriaceae dominated P1 (52.1%), P2 (91.1%), P4 (36%), and P5 (83.8%), while Pseudomonadaceae dominated all depths of P3 (94.8%). On the other hand, one patient showed a more balanced microbial profile, with several low-abundance families. The bacterial family composition and its variability across samples are illustrated in **Figure 4**.

**Figure 5:**
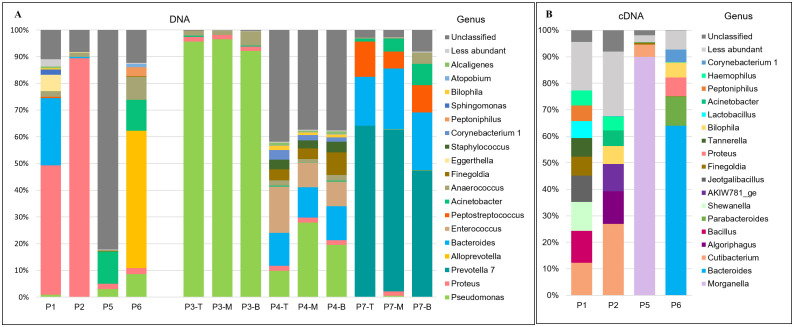
Relative abundance at genus level for **(a)** Total bacterial community of the seven patients with DFO and the **(b)** Ribosomally active bacterial community of the four patients with DFO. Samples P1, P2, P5, and P6 are single samples, one per patient. Samples P3, P4, and P7: three layers of bone tissue: Superficial or Top, Middle and Bottom.

At the genus level, on average, 37 ± 15 bacterial genera were identified per sample. Patient-specific dominant genera were observed, with *Proteus* (P3), *Pseudomonas* (P1–P2), and *Prevotella 7* (P7) predominating. The most prevalent Gram-negative aerobic genera were *Pseudomonas*, *Acinetobacter*, *Sphingomonas* and *Alcaligenes*. Notably, anaerobic bacteria predominated, accounting for 10/18 genera with the highest RA%, with *Anaerococcus* being the only genus detected in all samples, despite its low relative abundance (8.7%–0.3%). *Staphylococcus* (~3.5%), a facultative anaerobe, was detected only in patient P4. Overall, the coexistence of anaerobic, aerobic and facultative taxa underscores the polymicrobial nature of DFO (**Figure 5A**). Within the ribosomally active bacteria showing the highest RA%, *Cutibacterium* was present in all samples, although at low relative abundance (27% to 1.6 × 10^-2^%), whereas *Morganella* (90%) and *Bacteroides* (64%) were exclusively detected in P5 and P6, respectively. Consistent with metagenomic analysis, anaerobic genera (6/18) and facultative anaerobes (8/18) predominated over aerobic genera (2/18). Several genera, including *Bacteroides*, *Proteus*, *Finegoldia*, *Bilophila*, *Acinetobacter*, *Peptoniphilus* and *Corynebacterium*, were detected in both 16S rDNA- and 16S rRNA-derived datasets, indicating their presence in the total community as well as their association with the ribosomally active fraction (**Figure 5B**).

### 3.5 Microbiota composition by bone layer: genomic DNA analysis

To understand the spatial distribution of the microbiota in DFO, a gDNA-based metagenomic analysis was performed across bone depth layers (top, middle and bottom) for patients P3, P4, and P7. Six families were found exclusively in the superficial layer (Top), with Methylophilaceae being the most represented (>1%). The middle layer had the highest diversity, with 19 exclusive families, notably Fusobacteriaceae (>1%). In the bottom layer, 10 unique families were identified, including Micromonosporaceae as the most abundant (>1%). Several families were shared between layers: six between middle and bottom, and five between top and middle. Dominant families present across all layers included Enterobacteriaceae (52.5%), Family_XI (5.96%), Moraxellaceae (5.01%), Peptostreptococcaceae (4.66%), Enterococcaceae (3.95%) and Corynebacteriaceae (52.09%) (**Supplementary Figure S1**).

Samples from P3 (all depths) and P5 showed the most homogeneous microbial composition and strongest interconnections. P3 samples were closely related to P4 and P6, while P7 samples clustered more closely with P1 and P2, though sequence numbers in these were lower (**Supplementary Figure S2**).

At the genus level, samples from P3 were predominantly composed of *Pseudomonas* across all layers (>90%). In P4, *Pseudomonas* (~19.1%) was detected together with other unclassified Enterobacteriaceae (~34.2%), while *Prevotella* accounted for more than 60% of the community in P7 (**Figure 5A**). Consistently, as bone depth increased, the relative coverage of the most prevalent anaerobic genera also increased.

### 3.6 Ribosomally active microbiota in DFO

**Figure 6:**
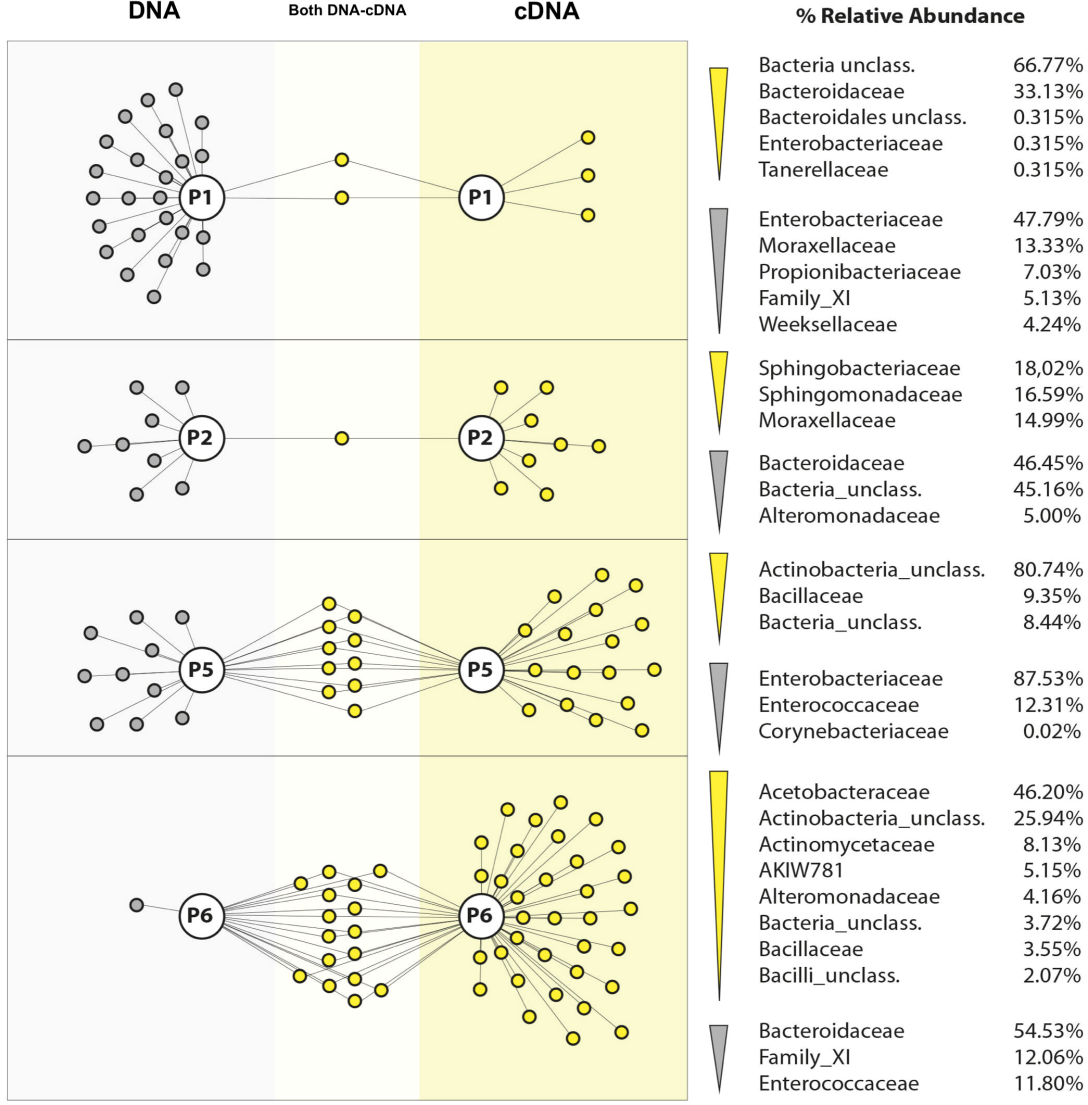
Relative abundance comparison between total bacterial and total ribosomally active bacterial communities in single samples with DFO.

Using cDNA sequencing of samples P1, P2, P5, and P6, ribosomally active bacterial at phylum and family levels were identified. The dominant phyla were: Proteobacteria (43.5%), Firmicutes (20.7%), Bacteroidetes (19.8%), Actinobacteria (13.6%), and Chloroflexi (9.4%) (Figure XA). The most abundant families included Enterobacteriaceae (18.5%), Bacteroidaceae (11.8%), Enterococcaceae (9.6%), Propionibacteriaceae (8.1%), Burkholderiaceae (4.6%), and others (Figure XB). At family level, Enterobacteriaceae (36.5%) and Enterococcaceae (28.3%) were the most ribosomally active, followed by Pseudomonadaceae (4.2%), Corynebacteriaceae (4.0%), Family_XI (2.9%), Lachnospiraceae (2.3%) and Bacteroidaceae (1.3%). Notably, Bacteroidaceae was the only family present in both total and active communities across all samples (**Figure 6**).

Comparisons between layer-specific and non-layered samples revealed that ribosomally active taxa were detected in all bone layers, although their distribution varied (**Supplementary Figure S1**). Some families were exclusive to the superficial/top (2), middle (3) or bottom (7) layers, while others were shared across two or all three layers. Overall, 67.8% of the taxa identified by 16S rRNA overlapped with those detected by 16S rDNA. The active-to-total ratio by layer was: 21/37 for superficial (top), 23/56 for middle, and 26/54 for bottom samples. Across all layers, the most abundant ribosomally active families were Enterobacteriaceae, Enterococcaceae, Corynebacteriaceae, Pseudomonadaceae, Family_XI, Lachnospiraceae, and Bacteroidaceae, suggesting their potential involvement in DFO-associated microbial activity.

Differences between DNA- and RNA-based profiles indicate that not all taxa detected at the DNA level are ribosomally active, while some taxa with low genomic abundance show increased ribosomal activity. Finally, most detected genera correspond to known cultivable bacteria, although several are fastidious or require strict anaerobic conditions; only one taxon (AKIW781_ge) represents an uncultured bacterial lineage identified exclusively by 16S rRNA gene sequencing.

## 4 Discussion

The predominant histopathological finding in our cohort was acute osteomyelitis, characterized by neutrophil infiltration, suppuration, presence of pyocytes, osteonecrosis, and bacterial colonies. Chronic osteomyelitis presented with bone marrow fibrosis and a plasma cell-rich infiltrate. The only case of chronic active osteomyelitis showed primary features of the acute form. It is worth noting that acute osteomyelitis may also include findings favoring infection, such as osteopenia, fibrosis, or trabecular bone fragmentation, suggesting a probable chronic ischemic process. Chronic osteomyelitis may result from immune-mediated mechanisms driven by less virulent microorganisms.

The polymicrobial nature is a hallmark of chronic infection disease, in which multiple bacterial species influence disease progression. High diversity indices (Shannon and Simpson) observed in most patients indicate a heterogeneous bacterial distribution, whereas lower richness indices (S_obs_ and Chao1) in several cases further support the polymicrobial nature of DFO in Chilean patients. These findings are consistent across all samples analyzed. Previous studies in French (Jneid et al., 2018) and Chinese (van Asten et al., 2016; Huang et al., 2022) patients also reported high microbial diversity in DFO, supporting its polymicrobial origin.

Conversely, in cases like P1 and P2, which exhibited low diversity but high richness, suggests infections dominated by a single bacterial taxon. In these instances, evidence points to disruption of the skin microbiome balance by pathogens adapted to this microenvironment (Huang et al., 2022).

Twelve bacterial phyla were identified, with Proteobacteria, Bacteroidetes, Firmicutes and Actinobacteria predominating, in qualitatively agreement with previous studies (Malone et al., 2017; Jneid et al., 2018). However, differences in RA%, suggest the influence of clinical and demographic factors on microbial composition in DFO. This variation likely reflects differences in infection patterns across healthcare facilities and the impact of geographic location on pathogen prevalence. Factors such as clinical management, patient occupation, and regional characteristics shape the dominant microbial profile, consistent with IWGDF recommendations emphasizing the importance of these factors in infection management (Senneville et al., 2024). Thus, our findings highlight the need for treatment approaches tailored to each clinical and locally context.

Although *S. aureus* is traditionally regarded as a primary DFO pathogen, and *Staphylococcus* spp. is the most prevalent reported genus in both culture- and sequencing- based studies, our results revealed a markedly lower prevalence (Shettigar et al., 2018; Hawkins et al., 2022; Lee et al., 2023; Chen et al., 2023; Morton and Coghill, 2024). Only 5% of DFO isolates correspond to *S. aureus*, and *Staphylococcus* spp. represented just 1% of all sequences from one single patient. In contrast, Gram-negative bacteria, particularly Proteobacteria and Bacteroidetes, were more prevalent. This shift in infection patterns suggests that conventional diagnostics focused on Gram-positive bacteria may be inadequately capture the full microbial landscape of DFO.

HTS-based strategies have significantly advanced our understanding of the diabetic foot microbiome, by enabling the detection of microorganisms present at low abundance or uncultivable under standard protocols (Malone et al., 2017; Jneid et al., 2018). In this study, the most genera detected by DNA- and RNA-based approaches correspond to known cultivable bacteria. The detection of the same genera in both DNA- and RNA-based analyses suggests that these taxa are not only present but also maintain ribosomal activity, it indicates that a given organism is not only viable but also potentially capable of a rapid response in a new environment (Emerson et al., 2017).

Notably, many of these taxa were strict anaerobes, including *Bacteroides*, *Bilophila*, *Finegoldia*, *Peptoniphilus*, *Anaerococcus*, *Eggerthella*, *Atopobium*, *Prevotella* and *Tannerella*. Their fastidious growth requirements likely explain their underrepresentation in routine diagnostics. Only one taxon, AKIW781_ge, represents an uncultured lineage detected exclusively by 16S rRNA gene sequencing.

The detection of these anaerobes in both DNA and ribosomal RNA fractions suggests that these taxa are not only present but also maintain ribosomal activity and underscores their potentially active role in the microbial community associated with DFO and biofilm persistence. Future work employing shotgun metatranscriptomic or proteomics is required to confirm the precise functional roles and metabolic activities of these highlighted taxa.

Low abundance, anaerobic or underestimated bacteria may play a “key role” in infection progression. Similar to other chronic biofilms-associated infections, microorganisms comprising a minor fraction of the community (0.001% of the total community) can modulate virulence and promote persistence, as demonstrated in periodontitis (Hajishengallis et al., 2012).

The detection ribosomally active Corynebacteriaceae across all bone layers underscores the relevance of molecular techniques in identifying emerging pathogens. *C. striatum*, increasingly associated with DFO, is a biofilm-forming and often multidrug-resistant organism (Qiu et al., 2023). While its role remains debated- ranging from benign to pathogen-it should not be dismissed as clinically irrelevant (Uçkay et al., 2014; Zheng et al., 2024).

The persistence of dominant families across the superficial (top), middle and bottom bone layers suggest that each patient harbors a unique, spatially structured infection-associated microbiome. This finding supports the rationale for individualized antimicrobial therapy, tailored to the patient-specific microbial profile, as proposed by previous studies (Jnana et al., 2020).

Despite the strengths of HTS-based approaches, this study has several limitations. These include the small sample size and challenges associated with patient recruitment, particularly given the invasive nature of bone sampling and the lack of direct clinical benefit for participants. Additionally, the limited size of bone specimens may have constrained the amount of genetic material available. Other considerations include the time required for analysis, associated costs, and the need for specialized multidisciplinary expertise. Finally, the quality and stability of extracted DNA, along with sequencing depth and threshold biases, may have influenced the detection of low-abundance microorganisms. These technical challenges should be considered when interpreting the results and highlight the need for further studies with larger sample sizes and standardized protocols to further validate these findings.

## 5 Conclusion

The results of this study support the polymicrobial etiology of DFO, characterized by a diverse microbial community influenced by clinical and geographic factors. Gram-negative bacteria were dominant, and some low-prevalence microorganisms may play a key role in infection dynamics. HTS revealed a complex microbial network, highlighting potentially underdiagnosed pathogens that may be overlocked by conventional diagnostic approaches.

Although current clinical guidelines in Chile rely on conventional culture-based methods for pathogen identification and antibiotic susceptibility testing, our findings indicate that these strategies could be substantially strengthened through integration with culture-independent molecular approaches. As sampling and analytical protocols become standardized, such combined methodologies may facilitate the identification of a core DFO microbiome and enable more personalized, targeted therapeutic strategies.

Given the heterogeneity of the microbial profiles observed across patients, the diagnostic limitations of conventional cultures and uncertainties about antibiotic susceptibility, empirical combination therapy-particulary regimen covering anaerobes, such as beta-lactams with beta-lactamase inhibitors, may be is recommended and justified in severe infections, with subsequent adjustments guided by antibiogram results.

Difficult-to-treat pathogens such as *C. striatum*, frequently overlooked in routine diagnostic and often associated with multidrug resistance-responsive only to vancomycin, linezolid, daptomycin, dalbavancin, tigecycline, or imipenem-warrant greater consideration in DFO diagnostic workflows. Their detection may have therapeutic implications, particularly in moderate to severe infections, where inadequate coverage could contribute to treatment failure or persistence (Silva-Santana et al., 2021).

Overall, integrating molecular diagnostics with conventional microbiology has the pontencial to improve diagnostic resolution and inform more effective therapeutic decision-making. A shift toward treatment strategies guided by the actual microbial landscape of DFO —rather than culture results alone—may contribute to improved outcomes and reduced rates of complications, including amputation.

## Data Availability

The data presented in this study are deposited in the NCBI Sequence Read Archive (SRA), BioProject accession number PRJNA1270973. The data are publicly accessible at https://www.ncbi.nlm.nih.gov/sra/PRJNA1270973.

## Supplementary material

Supplementary Figure 1 Distribution of bacterial families in different layers (Top, Middle and Bottom) of samples from patients with DFO, as determined by 16S rRNA sequencing (Illumina). **(A)** genomic DNA samples (P3, P4 and P7) and **(B)** RNA samples (P1, P2, P5 and P6).

Supplementary Figure 2 Microbial network showing OTU distribution at the family level from seven patients with DFO. Samples P1, P2, P5, and P6 are single samples, one per patient. Samples P3, P4, and P7: three layers of bone tissue (Top, Middle and Bottom).

## References

[B1] Acharya S. Soliman M. Egun A. Rajbhandari S. M. (2013). Conservative management of diabetic foot osteomyelitis. Diabetes Res. Clin. Pract. 101, e18–e20. doi: 10.1016/j.diabres.2013.06.010 23850116

[B2] Bashiardes S. Zilberman-Schapira G. Elinav E. (2016). Use of metatranscriptomics in microbiome research. Bioinf. Biol. Insights. 10, 19–25. doi: 10.4137/BBI.S34610 27127406 PMC4839964

[B3] Bauer A. W. Perry D. M. Kirby W. M. (1959). Single-disk antibiotic-sensitivity testing of staphylococci: an analysis of technique and results. AMA Arch. Internal Med. 104, 208–216. doi: 10.1001/archinte.1959.00270080034004 13669774

[B4] Bernard G. Pathmanathan J. S. Lannes R. Lopez P. Bapteste E. (2018). Microbial dark matter investigations: How microbial studies transform biological knowledge and empirically sketch a logic of scientific discovery. Genome Biol. Evol. 10, 707–715. doi: 10.1093/gbe/evy031 29420719 PMC5830969

[B5] Burillo A. Moreno A. Salas C. (2007). Diagnóstico microbiológico de las infecciones de piel y tejidos blandos. Enfermedades Infecciosas y Microbiología Clínica. 25, 579–586. doi: 10.1157/13111185 17953899

[B6] Charles P. G. Uçkay I. Kressmann B. Emonet S. Lipsky B. A. (2015). The role of anaerobes in diabetic foot infections. Anaerobe. 34, 8–13. doi: 10.1016/j.anaerobe.2015.03.009 25841893

[B7] Chen Y. Yang J. Wang Y. You J. Zhu W. Liu C. . (2023). Community-associated methicillin-resistant *Staphylococcus aureus* infection of diabetic foot ulcers in an eastern diabetic foot center in a tertiary hospital in China: a retrospective study. BMC Infect. Dis. 23, 652. doi: 10.1186/s12879-023-08631-z 37789270 PMC10548623

[B8] Choi Y. Banerjee A. McNish S. Couch K. S. Torralba M. G. Lucas S. . (2019). Co-occurrence of anaerobes in human chronic wounds. Microbial Ecol. 77, 808–820. doi: 10.1007/s00248-018-1231-z 30141127

[B9] Díaz-Velis L. Álvarez-Echeverría F. Garrido G. (2023) in Cultivo versus metagenómica para la identificación bacteriana en pacientes con osteomielitis de pie diabético: una revisión sistemática, 151, 206–221. (Revista Médica de Chile). Available online at: https://www.revistamedicadeChile.cl/index.php/rmedica/article/view/10152 (Accessed January 4, 2026). 10.4067/s0034-98872023000200206 38293856

[B10] Emerson J. B. Adams R. I. Román C. M. B. Brooks B. Coil D. A. Dahlhausen K. . (2017). Schrödinger’s microbes: Tools for distinguishing the living from the dead in microbial ecosystems. Microbiome. 5, 86. doi: 10.1186/s40168-017-0285-3 28810907 PMC5558654

[B11] Féron F. de Ponfilly G. P. Potier L. Gauthier D.-C. Salle L. Laloi-Michelin M. . (2021). Reliability and safety of bedside blind bone biopsy performed by a diabetologist for the diagnosis and treatment of diabetic foot osteomyelitis. Diabetes Care 44, 2480–2486. doi: 10.2337/dc20-3170 34475028

[B12] Game F. L. (2013). Osteomyelitis in the diabetic foot. Med. Clinics North America. 97, 947–956. doi: 10.1016/j.mcna.2013.03.010 23992902

[B13] Giurato L. Meloni M. Izzo V. Uccioli L. (2017). Osteomyelitis in diabetic foot: a comprehensive overview. World J. Diabetes. 8, 135–142. doi: 10.4239/wjd.v8.i4.135 28465790 PMC5394733

[B14] Gosalbes M. J. Durbán A. Pignatelli M. Abellán J. J. Jiménez-Hernández N. Pérez-Cobas A. E. . (2011). Metatranscriptomic approach to analyze the functional human gut microbiota. PloS One. 6, e17447. doi: 10.1371/journal.pone.0017447 21408168 PMC3050895

[B15] Hajishengallis G. Darveau R. P. Curtis M. A. (2012). The keystone-pathogen hypothesis. Nat. Rev. Microbiol. 10, 717–725. doi: 10.1038/nrmicro2873 22941505 PMC3498498

[B16] Hawkins B. K. Barnard M. Barber K. E. Stover K. R. Cretella D. A. Wingler M. J. B. . (2022). Diabetic foot infections: a microbiologic review. Foot. 51, 101877. doi: 10.1016/j.foot.2021.101877 35468387

[B17] Huang Y. Xiao Z. Cao Y. Gao F. Fu Y. Zou M. . (2022). Rapid microbiological diagnosis based on 16S rRNA gene sequencing: a comparison of bacterial composition in diabetic foot infections and contralateral intact skin. Front. Microbiol. 13. doi: 10.3389/fmicb.2022.1021955 36274710 PMC9582933

[B18] Jnana A. Muthuraman V. Varghese V. K. Chakrabarty S. Murali T. S. Ramachandra L. . (2020). Microbial community distribution and core microbiome in successive wound grades of individuals with diabetic foot ulcers. Appl. Environ. Microbiol. 86, e02608–e02619. doi: 10.1128/AEM.02608-19 31924616 PMC7054093

[B19] Jneid J. Cassir N. Schuldiner S. Jourdan N. Sotto A. Lavigne J. P. . (2018). Exploring the microbiota of diabetic foot infections with culturomics. Front. Cell. Infection Microbiol. 8. doi: 10.3389/fcimb.2018.00282 30155447 PMC6102383

[B20] Kalan L. R. Meisel J. S. Loesche M. A. Horwinski J. Soaita I. Chen X. . (2019). Strain- and species-level variation in the microbiome of diabetic wounds is associated with clinical outcomes and therapeutic efficacy. Cell Host Microbe. 25, 641–655.e5. doi: 10.1016/j.chom.2019.03.006 31006638 PMC6526540

[B21] Lam K. van Asten S. A. Nguyen T. La Fontaine J. Lavery L. A. (2016). Diagnostic accuracy of probe to bone to detect osteomyelitis in the diabetic foot: a systematic review. Clin. Infect. Dis. 63, 944–948. doi: 10.1093/cid/ciw445 27369321

[B22] Lee J. Mashayamombe M. Walsh T. P. Kuang B. K. P. Pena G. N. Vreugde S. . (2023). The bacteriology of diabetic foot ulcers and infections and incidence of Staphylococcus aureus small colony variants. J. Med. Microbiol. 72. doi: 10.1099/jmm.0.001716 37326607

[B23] Lin C. Liu J. Sun H. (2020). Risk factors for lower extremity amputation in patients with diabetic foot ulcers: a meta-analysis. PloS One. 15, e0239236. doi: 10.1371/journal.pone.0239236 32936828 PMC7494323

[B24] Macdonald K. E. Boeckh S. Stacey H. J. Jones J. D. (2021). The microbiology of diabetic foot infections: a meta-analysis. BMC Infect. Dis. 21, 770. doi: 10.1186/s12879-021-06516-7 34372789 PMC8351150

[B25] MacDonald A. Brodell J. D. Jr Daiss J. L. Schwarz E. M. Oh I. (2019). Evidence of differential microbiomes in healing versus non-healing diabetic foot ulcers prior to and following foot salvage therapy. J. Orthopaedic Res. 37, 1596–1603. doi: 10.1002/jor.24279 30908702 PMC6659747

[B26] Malone M. Johani K. Jensen S. O. Gosbell I. B. Dickson H. G. Hu H. . (2017). Next generation DNA sequencing of tissues from infected diabetic foot ulcers. EBioMedicine. 21, 142–149. doi: 10.1016/j.ebiom.2017.06.026 28669650 PMC5514496

[B27] Margozzini P. Passi Á. (2018) in Encuesta Nacional de Salud, ENS 2016-2017: un aporte a la planificación sanitaria y políticas públicas en Chile, Vol. 43. 30–34 (ARS medica). Available online at: https://www.arsmedica.cl/index.php/MED/article/view/1354 (Accessed January 4, 2026).

[B28] Morton K. E. Coghill S. H. (2024). *Staphylococcus aureus* is the predominant pathogen in hospitalised patients with diabetes-related foot infections: an Australian perspective. Antibiotics (Basel) 13, 594. doi: 10.3390/antibiotics13070594 39061276 PMC11273989

[B29] Neave M. Luter H. Padovan A. Townsend S. Schobben X. Gibb K. (2014). Multiple approaches to microbial source tracking in tropical northern Australia. Microbiology Open. 3, 860–874. doi: 10.1002/mbo3.209 25224738 PMC4263510

[B30] Parada A. E. Needham D. M. Fuhrman J. A. (2016). Every base matters: assessing small subunit rRNA primers for marine microbiomes with mock communities, time series and global field samples. Environ. Microbiol. 18, 1403–1414. doi: 10.1111/1462-2920.13023 26271760

[B31] Qiu J. Shi Y. Zhao F. Xu Y. Xu H. Dai Y. . (2023). The pan-genomic analysis of *Corynebacterium striatum* revealed its genetic characteristics as an emerging multidrug-resistant pathogen. Evolutionary Bioinf. 19, 11769343231191481. doi: 10.1177/11769343231191481 37576785 PMC10422898

[B32] Ruppé E. Lazarevic V. Girard M. Mouton W. Ferry T. Laurent F. . (2017). Clinical metagenomics of bone and joint infections: a proof of concept study. Sci. Rep. 7, 7718. doi: 10.1038/s41598-017-07546-5 28798333 PMC5552814

[B33] Schloss P. D. Westcott S. L. Ryabin T. Hall J. R. Hartmann M. Hollister E. B. . (2009). Introducing mothur: open-source, platform-independent, community-supported software for describing and comparing microbial communities. Appl. Environ. Microbiol. 75, 7537–7541. doi: 10.1128/AEM.01541-09 19801464 PMC2786419

[B34] Schmidt B. M. (2022). Emerging diabetic foot ulcer microbiome analysis using cutting edge technologies. J. Diabetes Sci. Technol. 16, 353–363. doi: 10.1177/1932296821990097 33576276 PMC8861789

[B35] Schmidt B. M. Erb-Downward J. Ranjan P. Dickson R. (2021). Metagenomics to identify pathogens in diabetic foot ulcers and the potential impact for clinical care. Curr. Diabetes Rep. 21, 26. doi: 10.1007/s11892-021-01391-7 34152440

[B36] Senneville É. Albalawi Z. van Asten S. A. Abbas Z. G. Allison G. Aragón-Sánchez J. . (2024). IWGDF/IDSA guidelines on the diagnosis and treatment of diabetes-related foot infections (IWGDF/IDSA 2023). Diabetes/Metabolism Res. Rev. 40, e3687. doi: 10.1002/dmrr.3687 37779323

[B37] Shannon P. Markiel A. Ozier O. Baliga N. S. Wang J. T. Ramage D. . (2003). Cytoscape: a software environment for integrated models of biomolecular interaction networks. Genome Res. 13, 2498–2504. doi: 10.1101/gr.1239303 14597658 PMC403769

[B38] Shettigar S. Shenoy S. Bhat S. Rao P. (2018). Microbiological profile of deep tissue and bone tissue in diabetic foot osteomyelitis. J. Clin. Diagn. Res. 12, DC20–DC22. doi: 10.7860/JCDR/2018/35462.11597

[B39] Silva-Santana G. Silva C. M. F. Olivella J. G. B. Silva I. F. Fernandes L. M. O. Sued-Karam B. R. . (2021). Worldwide survey of *Corynebacterium striatum* increasingly associated with human invasive infections, nosocomial outbreak, and antimicrobial multidrug-resistance 1976–2020. Arch. Microbiol. 203, 1863–1880. doi: 10.1007/s00203-021-02246-1 33625540 PMC7903872

[B40] Tönnies T. Rathmann W. Hoyer A. Brinks R. Kuss O. (2021). ‘Quantifying the underestimation of projected global diabetes prevalence by the International Diabetes Federation (IDF) Diabetes Atlas’. BMJ Open Diabetes Res. Care. 9, e002122. doi: 10.1136/bmjdrc-2021-002122 34400463 PMC8370495

[B41] Uçkay I. Gariani K. Pataky Z. Lipsky B. A. (2014). Diabetic foot infections: state-of-the-art. Diabetes Obes. Metab. 16, 305–316. doi: 10.1111/dom.12190 23911085

[B42] van Asten S. A. La Fontaine J. Peters E. J. Bhavan K. Kim P. J. Lavery L. A. (2016). The microbiome of diabetic foot osteomyelitis. Eur. J. Clin. Microbiol. Infect. Dis. 35, 293–298. doi: 10.1007/s10096-015-2544-1 26670675 PMC4724363

[B43] Villa F. Marchandin H. Lavigne J.-P. Schuldiner S. Cellier N. Sotto A. . (2024). Anaerobes in diabetic foot infections: pathophysiology, epidemiology, virulence, and management. Clin. Microbiol. Rev. 37, e00143–e00123. doi: 10.1128/cmr.00143-23 38819166 PMC11391693

[B44] Yazdanpanah L. Nasiri M. Adarvishi S. (2015). Literature review on the management of diabetic foot ulcer. World J. Diabetes. 6, 37–53. doi: 10.4239/wjd.v6.i1.37 25685277 PMC4317316

[B45] Zheng H. Na H. Yao J. Su S. Han F. Li X. . (2024). 16S rRNA seq-identified *Corynebacterium* promotes pyroptosis to aggravate diabetic foot ulcer. BMC Infect. Dis. 24, 366. doi: 10.1186/s12879-024-09235-x 38561650 PMC10986075

