# Correction: Metagenomic and ribosomal transcript profiles of diabetic foot osteomyelitis in Hispanic patients: underestimated bacteria in biofilm persistence

**DOI:** 10.3389/fcimb.2026.1902309

**Published:** 2026-06-17

**Authors:** Leonor Díaz-Velis, Francisco Salvador-Sagüez, Freddy Roach, Edgardo Mancilla, Marco A. Campos, Tay Ruiz-Gil, Mateo López-Moral, José Luis Lázaro-Martínez

**Affiliations:** 1 Laboratory of Applied Microbiology Research (LIMA), Faculty of Health Sciences, Universidad Católica de Temuco, , Temuco, Chile; 2 Molecular Ecology and Applied Microbiology Laboratory, Department of Pharmaceutical Sciences, Universidad Catolica del Norte, Antofagasta, Chile; 3 Infectious Diseases Unit, Dr. Leonardo Guzmán Regional Hospital, Antofagasta, Chile; 4 Department of Medical Sciences, Faculty of Medicine and Dentistry, Universidad de Antofagasta, Antofagasta, Chile; 5 Microbiology Area, Clinical Laboratory, Dr. Leonardo Guzmán Regional Hospital, Antofagasta, Chile; 6 Pathology Unit, Dr. Leonardo Guzmán Regional Hospital, Antofagasta, Chile; 7 Centro de Investigación, Innovación y Creación, Vicerrectoría de Investigación y Posgrado, Universidad Católica de Temuco, , Temuco, Chile; 8 Diabetic Foot Unit, Complutense University of Madrid, Madrid, Spain; 9 Independent Researcher, Antofagasta, Chile

**Keywords:** 16S rRNA gene sequencing, Biofilm, Bone microbiome, Diabetic foot osteomyelitis (DFO), Metagenomics, Polymicrobial infections, Ribosomal transcript analysis

In the **Acknowledgements**, the **Funding** source was displayed as FONDECYT Project No. 11280838. The correct statement is ANID-FONDECYT Project No. 1230308.

The original version of this article has been updated.

