# Supplementary figures and images for: Metagenomic and ribosomal transcript profiles of diabetic foot osteomyelitis in Hispanic patients: underestimated bacteria in biofilm persistence
Source: Front Cell Infect Microbiol. 2026 Jan 28;15:1729196. doi: 10.3389/fcimb.2025.1729196 (PMC12892099; doi:10.3389/fcimb.2025.1729196)

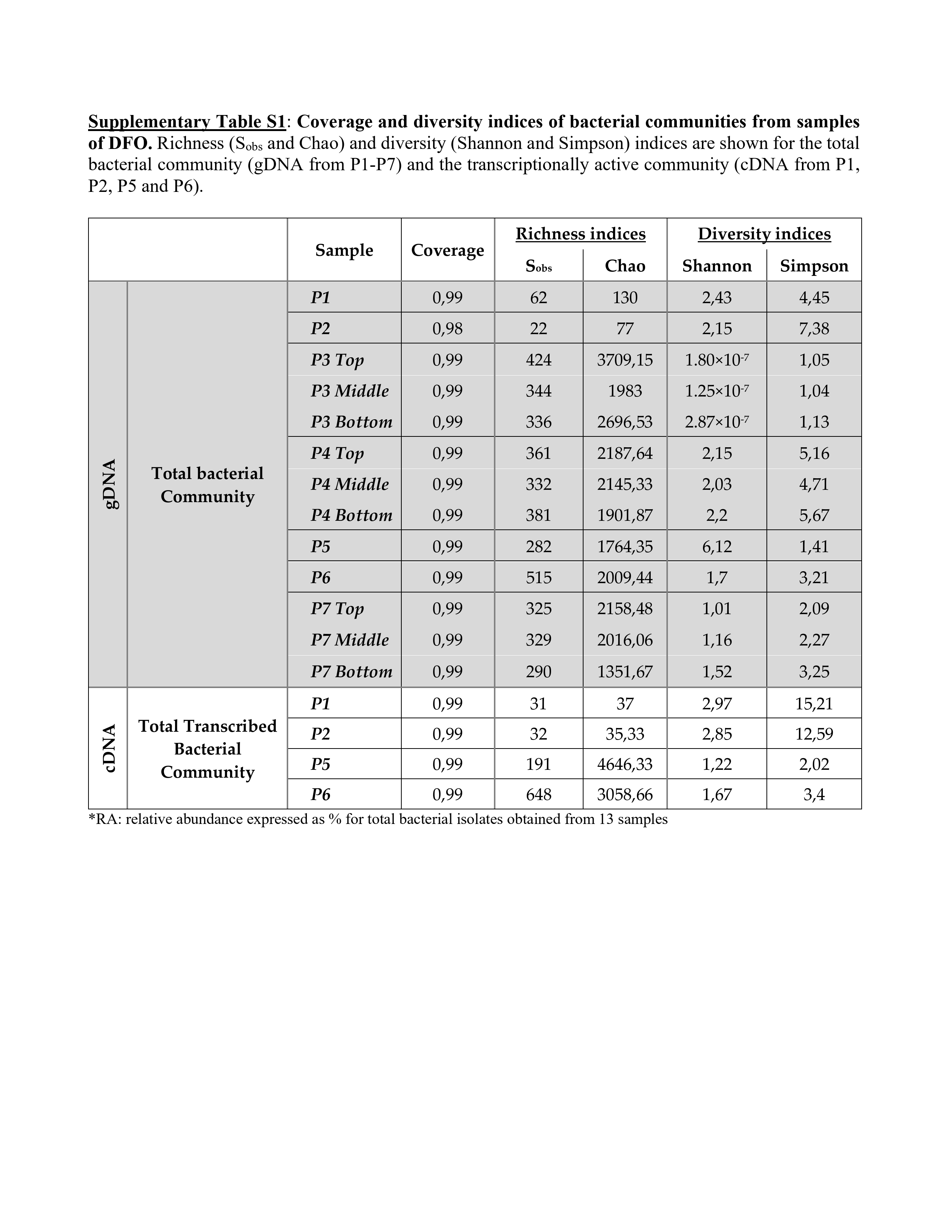

Supplement: Supplementary file 3 [file SupplementaryFile1.jpeg]
